# Enhanced Thermoelectric Performance of BST/WSe_2_ Heterostructures Through Defect‐Driven Microstructural Modifications

**DOI:** 10.1002/smtd.202501284

**Published:** 2025-10-06

**Authors:** Karan Giri, Yen‐Ling Wang, Yi‐Ting Wu, Chun‐Hua Chen

**Affiliations:** ^1^ Department of Materials Science and Engineering National Yang Ming Chiao Tung University Ta‐Hsueh Rd. 1001 Hsin‐Chu 30010 Taiwan R.O.C

**Keywords:** BST/WSe_2_ heterostructure, energy filtering, multiscale defects, pulsed laser deposition, thermoelectric

## Abstract

This study presents the fabrication of BST/WSe_2_ heterostructured films using dual‐beam pulsed laser deposition to enhance thermoelectric performance. Periodic incorporation of WSe_2_ into the BST matrix induces vertical perturbations that generate structural imperfections, such as staggered stacking, ripplocations, swapped bilayers, interlayer distortions, edge dislocations, and isolated W atoms, which collectively enhance phonon scattering and modulate charge transport. Additionally, the ambipolar nature of WSe_2_ facilitates the tuning of carrier concentration and energy filtering, thereby contributing to improved thermoelectric efficiency. These effects are more pronounced in films deposited at 573 and 673 K, where excessive defect scattering suppresses carrier mobility, lowering electrical conductivity and power factor. In contrast, films grown at 623 and 723 K achieve a better balance, where moderate defect scattering and thermal excitation help offset mobility losses. Notably, the 623 K sample achieves a power factor of ≈60.72 µW cm^−1^ K^−2^ at 447 K, exceeding previous reports. These findings underscore the potential of defect and interface engineering, when coupled with ambipolar semiconductor WSe_2_, for advancing the design of scalable, high‐performance thermoelectric materials.

## Graphical abstract

Structural and compositional design of BST/WSe_2_ heterostructures for improved thermoelectric performance.

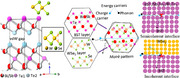

## 1 Introduction

The continuous release of waste heat from industrial processes and vehicles presents significant environmental challenges. To address these limitations, research efforts have focused on developing novel thermoelectric materials and enhancing existing ones to support sustainable energy harvesting and cooling technologies.^[^ ^1^, ^2^, ^3^ ^]^ Additionally, the design of cost‐effective power generation and solid‐state refrigeration devices has emerged as a focal area, emphasizing the need for high‐efficiency materials in heat‐to‐electricity conversion.^[^ ^4^, ^5^ ^]^ Among widely used thermoelectric materials, bismuth telluride (Bi_2_Te_3_) and its derivatives are state‐of‐the‐art materials known for their highly efficient conversion of low‐grade heat into green energy at room temperature through the Seebeck, Peltier, and Thomson effects.^[^ ^6^, ^7^, ^8^ ^]^ The energy conversion capability of thermoelectric materials is exceptionally reliable and depends on the dimensionless figure of merit, $ZT=\frac{\sigma S^{2}T}{\;k}\;$, where *T*, *k*, *σ*, and *S* are absolute temperature, thermal conductivity (sum of electronic and lattice contributions), electrical conductivity, and Seebeck coefficient, respectively. The practical application of these materials is limited by their low ZT value, explicitly influencing Carnot's efficiency as governed by the second law of thermodynamics. Enhancing thermoelectric performance requires a simultaneous increase in the power factor (PF) and reduction in thermal conductivity despite their interdependence through charge concentration.^[^ ^9^, ^10^ ^]^ Key factors for high‐efficiency thermoelectric materials include a high Seebeck coefficient, a moderate electrical conductivity, and a low thermal conductivity. We aim to optimize the trade‐off between *S* and *σ* by engineering multiscale microstructures and interfaces in the pulsed laser‐deposited thin film. These introduced functional structures potentially modulate charge transport and effectively scatter a broad spectrum of phonons, reducing thermal conductivity and enhancing overall thermoelectric performance.

A key approach to optimizing thermoelectric performance is to engineer structurally mismatched or twisted interfaces that facilitate selective carrier transport and enhance phonon scattering. Studies suggest that incorporating nanostructures into the host material enhances the power factor (PF) through quantum confinement effects while reducing thermal conductivity more than electrical conductivity. This is because phonons and charge carriers interact differently with internal interfaces due to their different scattering lengths.^[^ ^9^, ^11^ ^]^ Fabricating the material with micro‐ and nanostructural defects, such as grain boundaries, dislocations,^[^ ^12^ ^]^ mismatches,^[^ ^13^, ^14^ ^]^ planar twist,^[^ ^15^, ^16^ ^]^ and stacking faults,^[^ ^17^ ^]^ can create or modify diverse interfaces that enhance thermoelectric performance.^[^ ^18^, ^19^ ^]^ Pulsed laser deposition (PLD) is particularly well‐suited for this purpose, as it provides superior control over composition, thickness, and crystal structure, enabling the deposition of high‐quality thin films with tailored properties.^[^ ^20^, ^21^ ^]^ As a bottom‐up approach, it possesses precise stoichiometry control and diverse structural configurations that facilitate the incorporation of multiscale defects, further enhancing thermoelectric performance. For instance, the effectiveness of PLD is demonstrated in fabricating Bi_0.7_Sb_1.3_Te_3_ thin films, achieving a PF 25 µW cm^−1^ K^−2^ by incorporating multiple nanostructures that lead to coherent interfaces.^[^ ^22^ ^]^ A Bi_0.5_Sb_1.5_Te_3_ (BST) thin film grown at room temperature on a fused silica substrate reported a PF of ≈38 µW cm^−1^ K^−2^ at 380 K.^[^ ^23^ ^]^ Such findings underscore the potential of PLD for optimizing the thermoelectric properties through controlled defect engineering.

**Figure 1:**
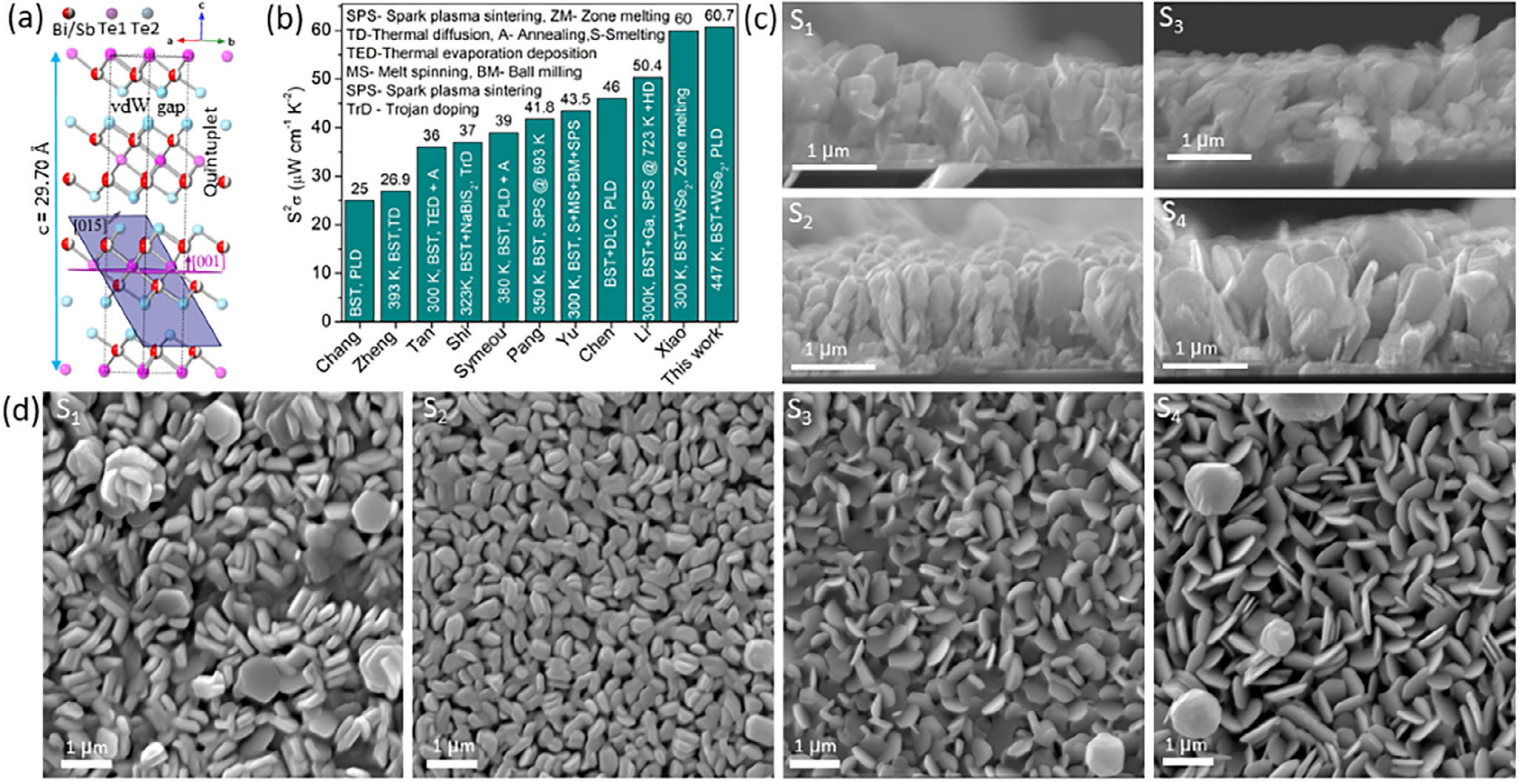
a) Edge‐on view of the crystallographic structure of BST, illustrating quintuple‐layer stacking and vdW gaps. The (*001*) and (*015*) planes are labeled, and the unit cell is outlined for clarity. b) Power factor comparison between pristine BST and composites with different additives. c) Cross‐sectional SEM images of four representative samples highlighting distinct microstructural features. d) Surface SEM micrographs revealing the morphology and topographic distribution of the deposited films.

The host material, bismuth antimony telluride, is a layered material with a quintuple atomic arrangement (Te_1_‐Bi/Sb‐Te_2_‐Bi/Sb‐Te_1_) (**Figure** **1**a) and rhombohedral Bravais lattice belonging to the R3̄m space group. The quintuples are stacked along the c‐axis through van der Waals (vdW) interactions. The vdW gap between adjacent Te_1_ atomic layers significantly influences thermoelectric properties by influencing charge transport and phonon scattering. In Bi_0.3_Sb_1.7_Te_3_ thin film fabricated via thermal diffusion, atomic diffusion within the vdW layers was successfully induced, resulting in a PF of ≈27 µW cm^−1^ K^−2^ at 393 K.^[^ ^24^ ^]^ Another study has shown that tuning the vdW gaps through alloying can optimize *n*, thereby improving *σ* while preserving a high *S*. This synergistic effect yields a PF of ≈49 µW cm^−1^ K^−2^ at 300 K.^[^ ^25^ ^]^ These advancements emphasize interface engineering as a powerful strategy for tuning transport properties in complex heterostructures. Extensive research has been conducted on pristine BST and BST‐based composites using various synthesis methods, with the reported PFs illustrated in Figure 1b.^[^ ^26^, ^27^, ^28^, ^29^ ^]^

The dopant material WSe_2_ (space group P6_3_/mmc), one of the transition‐metal dichalcogenides (TMDCs), consists of hexagonally arranged metal atom layers sandwiched between two chalcogen atom layers, with individual layers typically 6–7 Å thick (Figure 1a).^[^ ^30^ ^]^ In bulk form, it is an indirect bandgap semiconductor (1–1.3 eV), whereas in monolayer form, it transitions to a direct bandgap material (≈1.6 eV).^[^ ^31^ ^]^ Atomically thin sheets of WSe_2_ demonstrate high in‐plane carrier mobility and tunable conductance through electrostatic modulation. The ambipolar behavior of this dopant plays a pivotal role in this study, as its tunable Fermi level can be shifted toward either the valence or conduction band under an external electric field, enabling both hole and electron conduction. It exhibits ultra‐low thermal conductivity (≈0.05 W m^−1^ K^−1^)^[^ ^32^ ^]^ and exceptional electrical properties.^[^ ^33^ ^]^ Although WSe_2_ has been extensively studied for field‐effect transistors and light‐emitting diodes,^[^ ^34^, ^35^ ^]^ it remains underexplored in thermoelectrics.^[^ ^36^ ^]^ Its atomically thin structure enables quantum dot‐like defects^[^ ^37^ ^]^ that modulate electronic states and enhance carrier confinement. Encapsulating WSe_2_ within the BST matrix can generate multiple microstructures. Owing to its superior thermal stability^[^ ^38^ ^]^ and thermoelectric performance, WSe_2_ may enhance electrical transport while simultaneously reducing thermal conductivity.

This study reports the first successful incorporation of WSe_2_ into a p‐type Bi_0_._5_Sb_1_._5_Te_3_ matrix using PLD. Thin films were synthesized at four different substrate temperatures: 573, 623, 673, and 723 K, referred to as S1, S2, S3, and S4, respectively. We deposit a novel heterostructured, thin film composed of alternating regions of pure BST and BST/WSe_2_ composite, although distinct boundaries are not observed. The strategy is to create interfaces and defects that facilitate charge transport while suppressing phonon propagation. The heterostructure is stabilized by strong covalent bonding within the planes, and adjacent layers are held together by comparatively weak van vdW interactions. The resulting superlattice‐like architecture^[^ ^39^ ^]^ potentially enhances the Seebeck coefficient while maintaining moderate electrical conductivity, improving PF.

## 2 Results and Discussion

### 2.1 Morphology and Structural Characterization

The scanning electron microscopy (SEM) cross‐sectional (Figure 1c) and surface (Figure 1d) views reveal the morphology of the deposited thin films, highlighting the critical influence of substrate temperature on microstructural evolution. All samples exhibit uniform microstructures, with S3 and S4 displaying vertically aligned flake‐like structures extending to the substrate interface, indicating that the deposition conditions favor stable growth in this orientation. In the in‐plane direction, the flakes exhibit irregular orientation, and shadowing effects during deposition potentially contribute to the porous architecture. Notably, the higher substrate temperature in S4 promotes larger lateral flake dimensions and increased porosity. In contrast, S2 exhibits a dense columnar morphology, whereas the lower substrate temperature in S1 leads to a less defined and more irregular structure. Additionally, distinct bright surface features are attributed to isolated tungsten (W) atoms, likely resulting from selenium (Se) volatilization during ablation and elevated substrate temperatures.

Energy‐dispersive spectroscopy (EDS) mapping (Figure S1, Supporting Information) confirms uniform elemental distribution, indicating the high quality of the deposited thin films. Quantitative analysis (Table S1, Supporting Information) reveals slight compositional variations due to WSe_2_ incorporation into the BST matrix. All four samples exhibit relatively stable Te content, ensuring preservation of the anionic framework. The combined Bi and Sb content also remains nearly constant, consistent with the nominal Bi_0_._5_Sb_1_._5_Te_3_ stoichiometry. Nonetheless, slight variations in the Bi‐to‐Sb ratio could subtly affect the electronic structure. Minor deviations in total Bi+Sb and Te across samples suggest possible cation or anion vacancies, and the W and Se contents display more pronounced variability. S1 exhibits minimal WSe_2_ incorporation, with the lowest W (0.55 at%) and Se (0.14 at%) levels. S3 exhibits excessive W (7.97 at%) and relatively high Se (0.75 at%), which may potentially introduce structural distortions or defect‐related scattering. Meanwhile, S2 and S4 exhibit moderate and comparable concentrations of W (≈1.5 at%) and Se (≈0.6 at%), indicating a more controlled incorporation that may promote balanced charge transport and phonon scattering.

#### 2.1.1 XRD Analysis

**Figure 2:**
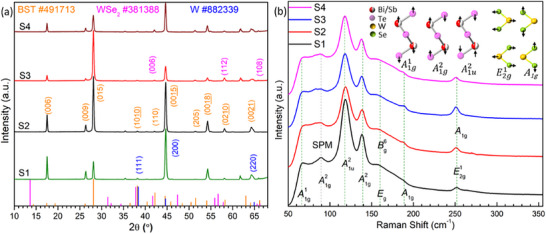
a) XRD patterns of the four deposited samples, illustrating phase identification and preferred crystalline orientations. For clarity, the PDF reference pattern intensities have been uniformly scaled by a factor of 300. b) Raman spectra of BST/WSe_2_ samples, with a schematic (top right) depicting the corresponding vibrational modes.

**Figure** **2**a demonstrates the θ–2θ XRD scan of four specimens within the Bragg angle range of 10°–70°, revealing dual preferred orientation along the (*001*) and (*015*) planes. This dual orientation originates from variations in film growth across the thickness, consistent with our earlier findings.^[^ ^22^, ^40^, ^41^ ^]^ The lower regions of the film favor (*001*) alignment, and the upper ones develop flake‐like structures with (*015*) orientation as the film thickens. The observed Miller indices and corresponding intensities align well with the standard powder diffraction patterns of Bi_0_._5_Sb_1_._5_Te_3_ (PDF #491713), WSe_2_ (PDF #381388), and W (PDF #882339), confirming rhombohedral BST as the primary phase, with hexagonal WSe_2_ and cubic W identified as secondary phases. Figure S2 (Supporting Information) presents the XRD peaks of each sample separately for comparison.

The consistent 2θ positions confirm structural stability, while the well‐defined and sharp peaks indicate high crystallinity.^[^ ^42^ ^]^ The W peaks arise due to Se volatility at elevated temperatures; however, weak or absent WSe_2_ peaks are attributed to its lower concentration resulting from its periodic ablation and reduced laser intensity. The diffraction peak marked with an asterisk at 62° corresponds to the WO_3_ phase (PDF #89‐8053), which is absent in the S2 sample, suggesting that the deposition conditions in S2 effectively suppress oxide formation.

Moreover, peak broadening at (*015*), (*1010*), and (*0015*) reflections further supports the presence of nanoscale structural features. Crystallite sizes, calculated using Scherrer's formula, range from 26–34 nm (S1), 29–38 nm (S2), 27–37 nm (S3), and 26–36 nm (S4), slightly smaller than those of pristine BST (32–44 nm, 32–45 nm, 28–43 nm, and 29–46 nm for S1–S4, respectively). Correspondingly, the average dislocation densities, 0.72 × 10^−3^ nm^−2^, 0.71 × 10^−3^ nm^−2^, 0.87 × 10^−3^ nm^−2^, and 0.75 × 10^−3^ nm^−2^, respectively (Table S2, Supporting Information), suggest a moderate increase in defect density in the heterostructures. Lattice parameter analysis (Table S3, Supporting Information) reveals a compressed structure in both pristine (a ≈ 4.21 Å, c ≈ 29.85 Å) and heterostructured BST (a ≈ 4.20 Å, c ≈ 29.70 Å) films compared to standard BST powder (a = 4.28 Å, c = 30.52 Å). This contraction is attributed to grain size effects in the thin films.^[^ ^43^ ^]^ The high surface‐to‐volume ratios and strain at grain boundaries can lead to inward lattice relaxation, resulting in overall compression. Additionally, the crystallite size in our films falls within the 25–45 nm range, the observed isotropic lattice contraction (in both a and c) aligns with the contraction regime reported in nanocrystalline solids, where surface‐tension‐induced hydrostatic pressure dominates.^[^ ^44^, ^45^ ^]^ In the heterostructures, a further slight reduction in lattice parameters is likely due to the d‐spacing mismatch between the WSe_2_ and BST layers,^[^ ^46^ ^]^ which induces additional lattice compression. Furthermore, the smaller out‐of‐plane lattice constant of WSe_2_ (c = 12.96 Å) may disrupt epitaxial alignment, influencing the nucleation and growth dynamics of the overlying BST layers and contributing to the observed strain behavior.

#### 2.1.2 Raman Analysis

Raman scattering provides insights into short‐range interactions, phonon vibrations, and local structural variations in BST/WSe_2_ thin films. The observed Raman peaks – $A_{1g}^{1}$ (66–69 cm^−1^ acoustic mode, Sb_2_Te_3_^[^ ^47^, ^48^ ^]^), $A_{1g}^{2}$ (88–90 cm^−1^, surface phonon mode (SPM) – Bi_2_​Te_3_^[^ ^49^ ^]^), $A_{1u}^{2}$ (118.77 cm^−1^ Bi_2_​Te_3_^[^ ^50^ ^]^) and $A_{1g}^{2}$ (≈138 cm^−1^, Sb_2_Te_3_^[^ ^51^ ^]^) – exhibit slight redshifts^[^ ^52^ ^]^ compared to literature values. These shifts arise from variations in the Sb/Bi ratio and laser excitation power (75 mW), which influence local vibrational modes and induce lattice strain.^[^ ^53^ ^]^ The vibrational mode $A_{1g}^{1}$ involves in‐phase vibrations of Bi/Sb and Te(1) atoms, whereas $A_{1g}^{2}$ exhibits out‐of‐phase motion. Overall, the spectra (Figure 2b; Table S3, Supporting Information) reflect features of both Bi–Te and Sb–Te bonding environments, indicating compositional and structural complexity within the BST matrix.

The reduced thickness weakens interlayer interactions, enhancing $A_{1g}^{2}$ intensity due to less constrained out‐of‐plane vibrations.^[^ ^49^ ^]^ Such thinning leads to an $A_{1g}^{2}$ mode at ≈90 cm^−1^, susceptible to defects, strain, and surface roughness. This surface phonon mode (SPM) reflects the localized effects of the Bi_2_Te_3_ phase and symmetry breaking at interfaces due to weak interlayer interactions. Similar to previously reported Raman‐forbidden and IR‐active modes,^[^ ^49^, ^54^ ^]^ $A_{1u}^{2}$ mode at 118.77 cm^−1^ indicates inversion symmetry breaking between two interfaces, likely caused by weak Bi–Te(1) bonding, defects, strain, or nanostructuring. The peak at 138 cm^−1^ corresponds to either the longitudinal $A_{1g}^{2}$ or transverse *E_g_* mode, depending on the local structural environment, which can be verified via polarization‐dependent Raman measurements. A new peak at 155 cm^−1^(*E_g_*, $B_{g}^{6}\;$ mode^[^ ^55^ ^]^ Sb_2_Te_3_ derivatives) along with a shoulder peak at 188.3 cm^−1^ (*A*_1*g*_) suggest anharmonic effects and structural complexity.

In TMDCs, interlayer interactions strengthen atomic restoring forces, resulting in redshifts, whereas stacking‐induced dielectric screening leads to blueshifts.^[^ ^56^ ^]^ As the material transitions from bulk to monolayer, the differentiation between the in‐plane $E_{2g}^{1}$ and out‐of‐plane *A*_1*g*_ modes becomes more evident; however, these modes appear nearly degenerate in bulk due to high symmetry.^[^ ^57^, ^58^, ^59^, ^60^ ^]^ In our samples, the $E_{2g}^{1}$ and $A_{g}^{1}$ modes appear at ≈251.23 cm^−1^, and the absence of $B_{2g}^{1}$ peak (≈304 cm^−1^)^[^ ^59^ ^]^ confirms the presence of a few‐layered WSe_2._ These features also suggest that symmetry breaking is likely caused by strain or lattice mismatch at the interfaces.

To sum up, the observed spectra span three frequency regions: low (<70 cm^−1^: $A_{1g}^{1}--Sb_{2}Te_{3}$), medium (70–120 cm^−1^: $A_{1g}^{2}$ and $A_{1u}^{2}--Bi_{2}Te_{3}$), and high (>120 cm^−1^: $E_{g},A_{1g}^{2}--Sb_{2}Te_{3}$, $A_{1g},B_{g}^{6}--Sb_{2}Te_{3},\mathrm{and}\;E_{2g}^{1},A_{1g}--\mathrm{WS}e_{2}$), reflecting phonon anharmonicity. Low‐frequency modes, particularly $A_{1g}^{1}$, influence lattice thermal conductivity reduction, whereas mid‐ and high‐frequency modes contribute to phonon scattering and interfacial interactions, including energy filtering of mid‐energy carriers, which affect transport properties.^[^ ^61^, ^62^ ^]^

#### 2.1.3 XPS Analysis

Figure S3 (Supporting Information) presents XPS spectra, revealing prominent characteristic peaks with high intensity and narrow full width at half maximum (FWHM), indicative of well‐defined chemical states. The broader peaks indicate the presence of multiple bonding environments, potentially due to local chemical inhomogeneity or surface oxidation. The Te 3d_5/2_ and 3d_3/2_ peaks at 572.3 and 582.7 eV (FWHM≈1‐1.8) correspond to the Te^2−^ state in Sb_2_Te_3_ and Bi_2_Te_3_ with a spin‐orbit splitting of (≈10.4 eV).^[^ ^63^, ^64^ ^]^ Additional lower‐intensity broader peaks at 573.28, 583.31, and 588.46 eV indicate the less ordered Te environments. Peaks at 529.06 eV (Sb 3d_5/2_) and 538.43 eV (Sb 3d_3/2_), with a splitting of 9.37 eV, are consistent with Sb^3+^ in Sb_2_Te_3_.^[^ ^65^ ^]^ In contrast, peaks at 530.93 and 540.38 eV correspond to oxidized Sb^3+^, likely due to surface oxidation forming Sb_2_O_3_, which can introduce interfacial traps affecting thermoelectric performance.

Furthermore, Bi_2_Te_3_‐related peaks at 157.51 eV (Bi 4f_7/2_) and 162.87 eV (Bi 4f_5/2_), corresponding to Bi^3+^ with a spin‐orbit splitting of 5.36 eV and narrow FWHM≈1. A peak at 167.76 eV (Se 3p_3/2_) is likely attributed to WSe_2_, and in the range 32–34 eV, attributed to Sb in Sb_2_Te_3_.^[^ ^65^, ^66^ ^]^ The peaks at 33.89 and 32.56 eV correspond to Sb 4d states, with a binding energy difference of 1.33 eV, which is slightly lower than the typical spin‐orbit splitting for Sb 4d_5/2_ and 4d_3/2_. The peaks at 41.55 eV (4d_3/2_) and 40.13 eV (4d_5/2_) correspond more closely to those of metallic Te and oxidized Te.

Distinguishing signals from background noise (Figure S4, Supporting Information) is challenging for trace or heterogeneously distributed materials. The limited Se content reduces W─Se bonding, resulting in excess W in multiple oxidation states, contributing to the observed peak broadening and shifts in the XPS spectra. Deconvoluted peaks at 256.88 and 242.08 eV correspond to W 4d_3/2_ and 4d_5/2_, respectively, with a 14.80 eV splitting exceeding typical spin‐orbit splitting values, indicating altered chemical environments. Additional peaks at 251.49, 246.83, and 260.99 eV (FWHM≈5.5) suggest secondary interactions. These features likely arise from tungsten atoms occupying the vdW gaps, as confirmed by spherical aberration‐corrected transmission electron microscopy (Cs‐STEM). The weak or incomplete bonding of these atoms perturbs the local electronic environment, resulting in atypical XPS signatures.^[^ ^67^ ^]^

#### 2.1.4 Transmission Electron Microscopy

**Figure 3:**
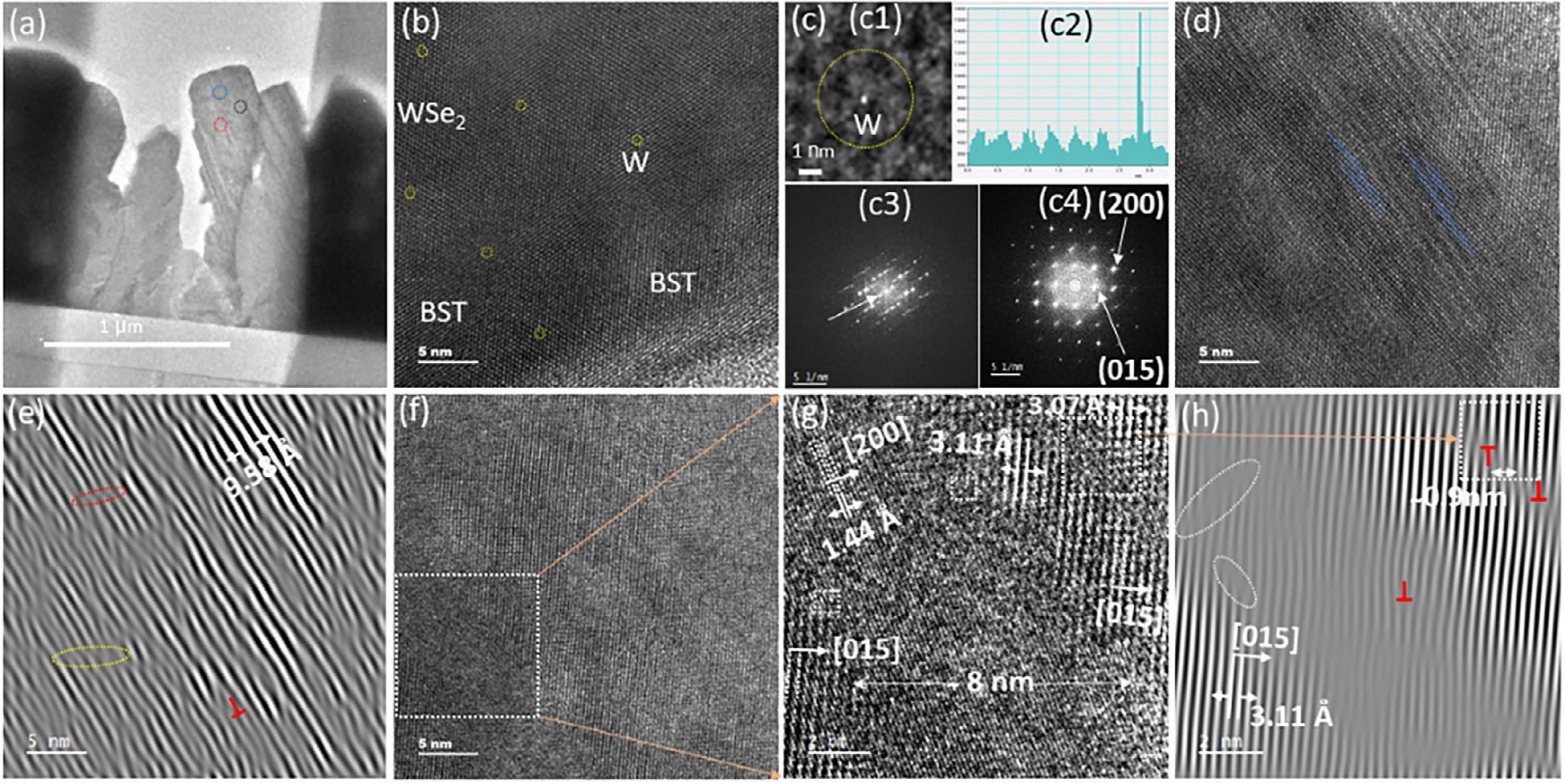
Microstructural analysis of BST/WSe_2_ thin films using Cs‐STEM. a) Bright‐field low‐resolution Cs‐STEM image showing flake‐like columnar microstructures; blue, red, and black circles indicate regions selected for high‐resolution analysis. b) High‐resolution Cs‐STEM image of the blue‐circled region in (a), revealing localized *moiré* patterns and isolated W atoms. c) Magnified view (c1) of a W atom (highlighted in yellow in b) and its corresponding line profile (c2). d) High‐resolution image of the red‐circled region in a), showing staggered BST atomic planes. e) IFFT image obtained by masking the Bragg diffraction spot (arrow in c3), highlighting the swapped bilayers (red ellipse) and ripplocation (yellow ellipse), edge dislocations (red), and locally increased interplanar spacing. f) Cs‐STEM micrograph of the black‐circled region in (a), showing BST as the dominant phase outside the boxed area and coexisting BST and WSe_2_ phases within. g) Magnified view of the boxed region in (f), displaying a phase boundary with semicoherent and incoherent interfaces; misfit regions are boxed. h) IFFT image derived by masking the (*015*) diffraction spot in FFT (c4), showing edge dislocations (red) and misfit regions (outlined in white).

**Figure 4:**
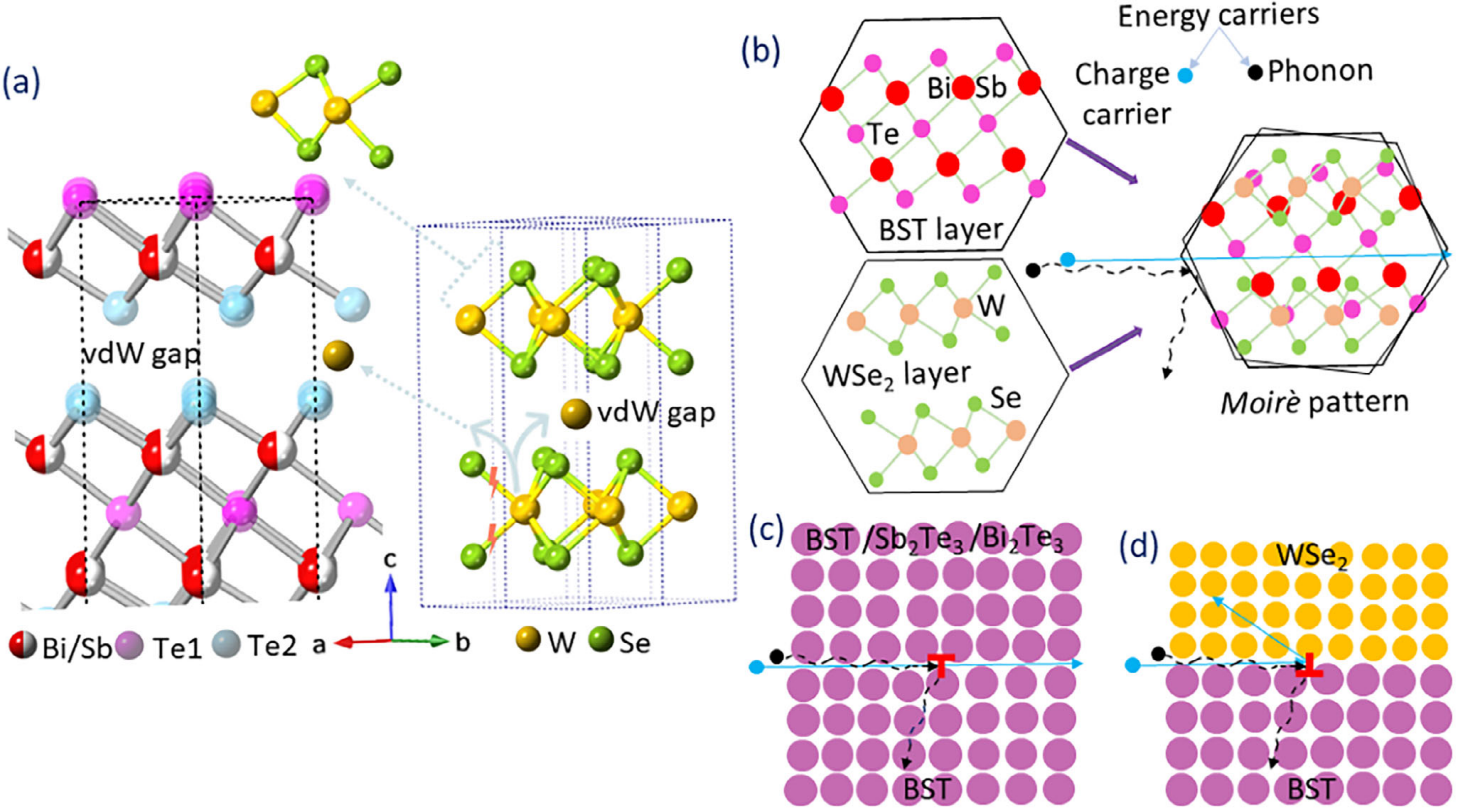
Schematic representation of structural and interfacial features affecting carrier transport. a) Formation of the WSe_2_ layer on the BST matrix during deposition; subsequent selenium evaporation results in isolated tungsten atoms occupying the vdW gaps. Dotted lines indicate the unit cells (partially shown for BST) for clarity. b) Moiré pattern formation due to twisting angles or misalignment between BST and WSe_2_ layers. c) Semicoherent interface: Lattice mismatch between different phases or domains with varying orientations induces edge dislocations (red). d) Incoherent interface: Strain at phase boundaries generates edge dislocations (red) due to poor lattice matching.

Cs‐STEM of sample S2 reveals multiscale defects that may influence transport properties. The observations from **Figure** **3** are schematically represented in **Figure** **4**. Three regions encircled in a low‐magnification TEM image (Figure 3a) are used to generate high‐resolution TEM micrographs. Based on the structure and average atomic number contrast, where image contrast is proportional to the square of the atomic number, the brighter layered regions correspond to the BST or Bi_2_Te_3_ phase, and the darker ones are likely the Sb_2_Te_3_ phase (Figure 3b). In a darker area with a well‐ordered hexagonal lattice, the WSe_2_ phase is identified, where the bright spots correspond to W atoms. Furthermore, isolated W atoms, likely resulting from Se evaporation, are observed to migrate into the vdW gaps, as shown in Figure 3c1 and corroborated by the line profile in Figure 3c2. Initially, W forms a WSe_2_ layer on top of the BST; however, following Se loss, the unbonded W atoms become destabilized and diffuse into the vdW gaps, as schematically illustrated in Figure 4a. Moreover, overlapping and twisting these layered phases generate local *moiré* patterns,^[^ ^15^ ^]^ resulting in interlayer coupling. This coupling acts as an energy filter, preferentially allowing high‐ and mid‐energy charge carriers to pass through, depending on the strength of the *moiré* potential barrier, while simultaneously scattering phonons. The schematic illustrating these phenomena is showcased in Figure 4b. These effects potentially elevate the average carrier energy, enhancing the Seebeck coefficient. Although some reduction in electrical conductivity can occur due to carrier scattering, a moderate conductivity level is expected to be achievable with appropriately engineered interfaces.

Figure 3d presents defects, including extensive staggered stacking faults^[^ ^17^ ^]^ (blue) in the BST layer, with intensity variations reflecting atomic number differences. The (Bi/Sb) – Te antisite defects disrupt the regular vdW structure, causing bilayers to split and merge with adjacent units, thereby altering the typical Te – (Bi/Sb) – Te – (Bi/Sb) – Te stacking sequence. These antisite defects, in which Bi or Sb atoms replace Te, expose Bi/Sb atoms that interact with neighboring Te layers, forming Te–Te interactions across vdW gaps and inducing structural mismatches that result in staggered stacking faults. The corresponding Fast Fourier Transform (FFT) and inverse FFT (IFFT) patterns are demonstrated in Figure 3c3,e, respectively. Strained atomic planes observed in the IFFT image are attributed to ripplocation (yellow), swapped bilayers (red), and local lattice distortions.^[^ ^68^ ^]^ These structural irregularities are accompanied by a locally increased interplanar spacing of 9.58 Å, suggesting strain relaxation in the affected regions.

Figure 3f illustrates the different phases and orientations within the matrix, which are further clarified through magnified views and IFFT patterns in Figure 3g,h, respectively. A lattice mismatch^[^ ^14^ ^]^ of ≈1.3% is observed between (*015*) planes of BST or possibly Sb_2_Te_3_ (with *d‐*spacing of 3.07 and 3.11 Å), which induces compressive strain, resulting in misfit or edge dislocations (highlighted in red in Figure 3h). This generates a semicoherent interface^[^ ^69^ ^]^ (schematically shown in Figure 4c) with a small lattice mismatch of narrow width (≈9 Å), facilitating more efficient charge transport by presenting a lower energy barrier than incoherent interfaces. In contrast, the incoherent interfaces between BST and the ≈80 Å‐wide WSe_2_ phase, with a significant lattice mismatch of 54%, introduce higher energy barriers that enhance the Seebeck coefficient via energy filtering while limiting electrical conductivity.

### 2.2 Thermoelectric Transport Properties

**Figure 5:**
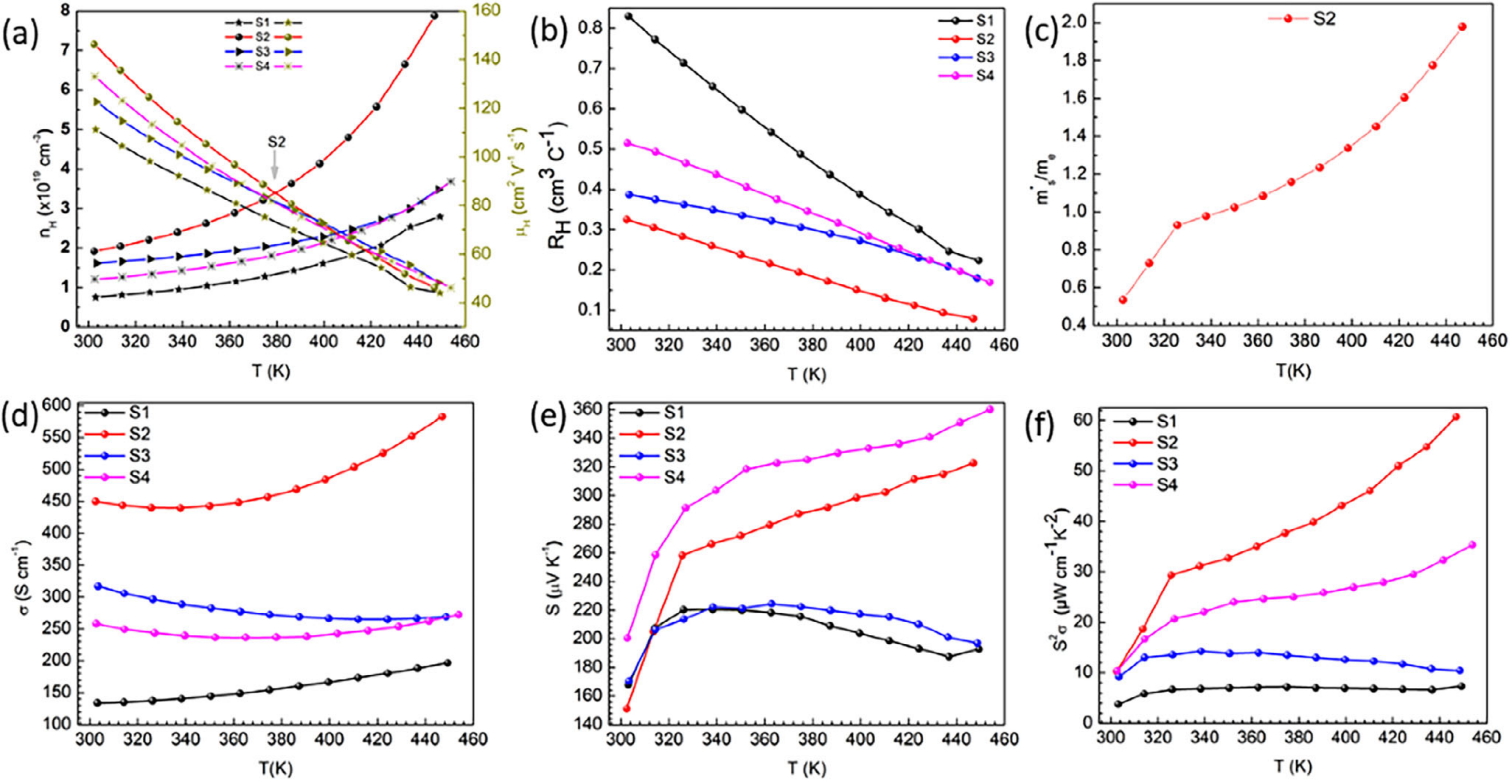
Temperature‐dependent transport properties of BST/WSe_2_ thin films: a) Hole concentration and mobility as a function of temperature. b) Average Hall coefficient across the measured temperature range. c) Seebeck effective mass versus temperature. d) Electrical conductivity as a function of temperature. e) Seebeck coefficient versus temperature. f) Power factor as a function of temperature, illustrating the combined effect of electrical conductivity and Seebeck coefficient.

**Figure** **5** illustrates the thermoelectric transport behavior of four samples as a function of temperature in the range of 300 to 455 K. The Hall mobility (µ_*H*_) of all samples decreases monotonically with temperature (≈146 to 46 cm^2^ V^−1^ s^−1^ for S2) (Figure 5a), following a power law µ_*H*_ *∝ T^−m^* with *m* = 2.33, 2.90, 2.23, and 2.55 for S1, S2, S3, and S4, respectively.^[^ ^70^, ^71^, ^72^ ^]^ The exponent *m*, derived from logarithmic plots (Figure S5, Supporting Information), exceeds the typical range for deformation potential scattering (1–1.5 for degenerate to nondegenerate semiconductors^[^ ^73^ ^]^), suggesting enhanced scattering mechanisms due to multiscale defects, described in the previous section. The crossover point indicated by an arrow for S2 marks a transition in transport behavior, occurring at a carrier concentration of ≈1.92–7.88 × 10^19^ cm^−3^, which falls within the range typically associated with optimized thermoelectric performance in similar systems.^[^ ^74^, ^75^ ^]^

Since Hall mobility is given by µ_*H*_ = |*R_H_* |*σ*, (µ_*H*_ =  µ, when the Hall factor *r_H_* = 1) the presence of thermally excited minority carriers reduces the Hall coefficient (*R_H_*), causing µ_*H*_ to decline more rapidly with temperature than the actual carrier mobility.^[^ ^73^ ^]^ In Figure 5b, the sharper decrease in |*R_H_* |for S1 exhibits this effect, indicating the dominant influence of defect‐driven scattering and minority carrier excitation on charge transport. The smaller slope observed for S2 (from 0.33 to 0.08 cm^3^ C^−1^) corresponds to an increase in carrier concentration from ≈1.9 × 10^19^ cm^−3^ to ≈7.8 × 10^19^ cm^−3^ (n = 1/qR_H_), indicating dominance of majority carriers and a reduced contribution from thermally activated minority carriers. Additionally, the Seebeck effective mass ($m_{s}^{*}$) is estimated using the single parabolic band model, $S=\frac{8\pi^{2}k_{B}^{2}T}{3eh^{2}}\;m_{s}^{*}{\left(\frac{\pi }{3n_{h}}\right)}^{2/3}$, where *k_B_* is the Boltzmann constant, *h* is the Planck constant, *n_h_* is the hole concentration, and e is the electron charge.^[^ ^76^ ^]^ Referring to Figure 5c for S2 (Figure S6, Supporting Information for all samples), the extracted Seebeck effective mass, calculated using the SPB model, increases from ≈0.5 m_e_ to 2 m_e_, exhibiting a linear trend between 300–330 K and a more rapid, parabolic‐like rise at higher temperatures. The smaller effective mass (≈0.5 m_e_) at lower temperatures and carrier concentrations indicates that transport is dominated by a single, nearly parabolic band valley. At higher temperatures or concentrations, the increase in effective mass reflects deviations from ideal parabolic behavior, likely arising from band non‐parabolicity and the possible onset of multivalley transport. However, the presence of multiple valleys cannot be confirmed solely by the increase in $m_{s}^{*}\;$ and requires further evidence, such as from electronic band structure^[^ ^77^ ^]^ or temperature‐dependent Hall factor analysis.^[^ ^78^ ^]^ The increasing $m_{s}^{*}$ and a steep decrease in µ_*H*_ indicate that defect scattering is occurring. The simultaneous increase in $m_{s}^{*}$ ​ and sharp decrease in µ_*H*_​ can be rationalized by defect scattering. An increase in effective mass typically reflects band distortion due to defects, while the reduced mobility indicates that carriers experience stronger scattering from these defects. Since phonon scattering alone is insufficient to account for such a pronounced drop in µ_*H*_, combined trend is more convincingly attributed to defect‐related scattering mechanisms. A comparative analysis of pristine BST and BST/WSe_2_ thin films is provided in the Figures S7–S10 (Supporting Information).

In the BST/WSe_2_ heterostructure, BST is a p‐type semiconductor owing to antisite defects and vacancies, whereas WSe_2_, though ambipolar,^[^ ^79^, ^80^ ^]^ typically exhibits n‐type behavior due to selenium vacancies. The reduced conductivity at lower temperatures is attributed to electron–hole recombination. At elevated temperatures, thermally excited electrons from WSe_2_ diffuse into the BST layer and recombine with majority carrier holes, reducing the net carrier concentration. With further temperature increase, thermal generation outweighs recombination losses, enhancing conductivity. This interplay between charge transfer and interfacial recombination governs the transport behavior in BST/WSe_2_ heterostructures.

In Figure 5d, the sample S1 exhibits intrinsic or lightly doped extrinsic semiconductor behavior, with electrical conductivity rising from 134 at 303 K to 197 S cm^−1^ at 449 K due to thermal carrier excitation. The S2 initially exhibits a slight drop in conductivity from 450 to 440 S cm^−1^ due to enhanced carrier scattering, followed by an increase to 583 S cm^−1^, reflecting a balance between carrier generation and scattering mechanisms. This behavior is characteristic of heavily doped, nondegenerate semiconductors approaching degeneracy,^[^ ^81^ ^]^ and the enhancement is attributed to an optimized carrier concentration, consistent with the Drude‐Sommerfeld free electron model (*σ* = *neµ*). S3 demonstrates a decline from 317 to 265 S cm^−1^ at 424 K, followed by a slight increase to 269 S cm^−1^ at 449 K, suggesting dominant scattering at lower temperatures, which is partially offset by carrier generation at higher temperatures. Similarly, S4 initially falls from 258 to 236 S cm^−1^ at 365 K, then rises to 273 S cm^−1^ at 454 K as thermal excitation compensates for scattering losses. These trends suggest that the ambipolar WSe_2_ and multiscale structural imperfections primarily suppress carrier mobility, significantly affecting carrier concentration in S1 and S3, and have a moderate effect in S2 and S4. These observed electrical conductivities are notably lower than previously reported values,^[^ ^13^, ^25^, ^27^, ^33^ ^]^ and this degradation is attributed to electron–hole recombination and enhanced scattering.

Hall (Figure 5b) and Seebeck (Figure 5e) coefficients exhibit positive values, confirming p‐type behavior and hole‐dominated transport. The relatively sharp increase in the Seebeck coefficient up to 320 K is attributed to energy filtering or carrier localization at defects, interfaces, or grain boundaries that preferentially enhance high‐energy carrier transport.^[^ ^82^ ^]^ A distinct ≈8 nm WSe_2_ region confined between BST phases (Figure 3g), indicating a quasi‐quantum well structure, may further support this filtering effect. Due to their nanoscale thickness and potential band offsets, spatially localized heterointerfaces likely contribute to energy filtering via quantum confinement effects, further influencing the thermoelectric properties.^[^ ^83^ ^]^

Samples S1 and S3 display a moderate increase in the Seebeck coefficient, rising from ≈168 to ≈220 µV K^−1^ at 339 K and from ≈170 to ≈225 µV K^−1^ at 363 K, respectively. By contrast, S4 exhibits a pronounced enhancement, increasing from ≈200 to ≈360 µV K^−1^ at 454 K. Notably, S2 shows the most remarkable growth, from ≈151 to ≈323 µV K^−1^ at 447 K, surpassing previously reported values.^[^ ^13^, ^24^ ^]^ This enhancement may arise from structural mismatching, isolated W atoms, and interfaces that introduce localized states or energy barriers promoting energy filtering. These barriers preferentially allow high‐energy carriers to contribute to transport, thereby enhancing the *S* and optimizing *n_h_*. Additionally, the absence of significant Seebeck suppression at elevated temperatures suggests minimal bipolar conduction in the BST/WSe_2_ system.

The PF versus temperature curves (Figure 5f) reflect these trends. S1 exhibits the lowest PF (7.17 µW cm^−1^ K^−2^) due to its moderate *S* and lower *σ*. S3 has PF 14.26 µW cm^−1^ K^−2^, indicating improved performance but limited efficiency. Despite exhibiting the highest *S* (360 µV K^−1^), S4 achieves a PF of 35.34 µW cm^−1^ K^−2^. S2 demonstrates the highest PF 60.72 µW cm^−1^ K^−2^ at 447 K, attributed to the best trade‐off between *σ* and *S*. Additionally, the energy‐filtering approach introduces barriers that maintain *σ*, while enhancing *S* more than compensates for this, leading to an overall increase in the PF. The observed PF for S2 is significantly higher than most previously reported values for pristine and doped BST materials (Figure 1b).^[^ ^22^, ^26^, ^27^, ^28^, ^29^, ^84^ ^]^ It surpasses the PFs 30 µW cm^−1^ K^−2^ reported for the in situ grown MoSe_2_ second phase in an n‐type bismuth telluride matrix with a semi‐common lattice interface,^[^ ^85^ ^]^ and 49 µW cm^−1^ K^−2^ for Sb_2_Te_3_/MoS_2_ multilayer structures.^[^ ^47^ ^]^ Although slightly lower than the 85 µW cm^−1^ K^−2^ reported for bilayer MoS_2_,^[^ ^86^ ^]^ the observed PF demonstrates a favorable trade‐off between *S* and *σ*, positioning the BST/WSe_2_ material as a strong candidate for thermoelectric applications.

## 3 Conclusion

This study demonstrates the successful fabrication of BST/WSe_2_ heterostructures using PLD, emphasizing the role of lattice mismatch and defect‐driven modifications in enhancing thermoelectric performance, as indicated by the compressed lattice parameters (a ≈ 4.20 Å, c ≈ 29.70 Å). Structural and morphological analyses confirm the formation of highly crystalline thin films with tunable defect densities across samples S1 to S4. Among these, sample S2, grown at 623 K, exhibits a broad lattice mismatch (1.3%–52%), which promotes the formation of multiscale defects. Such structural features modulate phonon scattering and charge transport, while simultaneously introducing effective energy‐filtering centers that collectively enhance thermoelectric performance. Additionally, isolated W atoms and ambipolar WSe_2_ regions modify the local electronic structure, enabling optimized carrier concentration. Transport measurements reveal that S1 and S3 exhibit lower performance, S4 shows moderate efficiency, and S2 achieves the best trade‐off among thermoelectric parameters. Specifically, S2 displays a carrier concentration of 1.92–7.88 × 10^19^ cm^−3^, moderate electrical conductivity (≈583 S cm^−1^), and a high Seebeck coefficient (323 µV K^−1^), resulting in a remarkable power factor of ≈60.72 µW cm^−1^ K^−2^ at 447 K, surpassing previously reported values. These findings highlight the synergistic effects of defect modulation and interface design, establishing ambipolar WSe_2_ as a promising additive in the BST system.

## 4 Experimental Section

### Thin Film Deposition

### Thin Film Deposition—Thin Film Growth

Composite thin films were deposited on (001) SiO_2_/Si substrates using dual‐beam pulsed laser deposition (PLD). BST (99.99% purity) and WSe_2_ (99.80% purity) powders were pressed into dense disc‐shaped targets. The ablation was performed in a chamber with a base pressure of 2 × 10^−5^ Torr and an Ar deposition pressure of 0.5 Torr, using a Q‐switched Nd:YAG laser (355 nm wavelength, 10 Hz repetition rate, 5 ns pulse width, ≈8.84 J cm^−2^ fluence). A beam splitter was employed to deliver ≈30% of the beam to one target and ≈70% to the other.

The films were grown in a multilayer sequence consisting of 11 alternating layers: BST as the first (bottom) layer, followed by BST + WSe_2_ composite, then BST, and so on, up to the topmost BST layer. The primary BST target was continuously ablated for 55 min, while the WSe_2_ target underwent periodic ablation cycles of 5 min to achieve controlled incorporation. Although an alternating multilayer design was intended, the interfaces between consecutive BST and BST + WSe_2_ layers were not atomically sharp, resulting in partially intermixed boundaries.

### Characterization

Sample morphology and composition were analyzed using field‐emission scanning electron microscopy (FESEM, JEOL JSM‐7800F) equipped with energy‐dispersive X‐ray spectroscopy (EDS, OXFORD MAX150). Structural analysis and phase identification were performed using X‐ray diffraction (XRD, Bruker AXS D8 Discover) with Cu Kα radiation (λ = 1.54 Å). Specimens for spherical aberration‐corrected transmission electron microscopy (Cs‐STEM) were prepared using a focused ion beam system (FIB; Hitachi NX2000), and high‐resolution images were acquired using a Cs‐corrected TEM (STEM023300). Raman spectroscopy was conducted using a micro‐Raman spectrometer (Horiba Jobin Yvon HR800UV) with a 532 nm diode laser. X‐ray photoelectron spectroscopy (XPS) was carried out using an Al Kα source (EM 0000012300) with a 650 µm spot size, 20.0 eV pass energy, and 0.100 eV step size over 171 steps. In‐plane transport coefficients were measured using a LINSEIS L79 HCS 1 under a 0.7 T magnetic field in the 300–475 K temperature range.

## Conflict of Interest

The authors declare no conflict of interest.

## Supporting information



Supporting Information

## Data Availability

The data that support the findings of this study are available from the corresponding author upon reasonable request.
