# Supplementary material for: Enhanced Thermoelectric Performance of BST/WSe2 Heterostructures Through Defect‐Driven Microstructural Modifications
Source: Small Methods. 2025 Oct 6;9(11):e01284. doi: 10.1002/smtd.202501284 (PMC12641349; doi:10.1002/smtd.202501284)
Supplement: Supplementary file 1 — Supporting Information [file SMTD-9-e01284-s001.docx]

Supporting Information

**Enhanced Thermoelectric Performance of BST/WSe₂ Heterostructures through Defect-Driven Microstructural Modifications**

*Karan Giri, Yen-Ling Wang, Yi-Ting Wu, and Chun-Hua Chen**

Department of Materials Science and Engineering, National Yang Ming Chiao Tung University, Ta-Hsueh Rd. 1001, 30010, Hsin-Chu, Taiwan, R.O.C.

[karangiri575@gmail.com](mailto:karangiri575@gmail.com), [karangiri575.c@nycu.edu.tw](mailto:karangiri575.c@nycu.edu.tw), [chunhuachen@nycu.edu.tw](mailto:chunhuachen@nycu.edu.tw)

**Lattice distortion analysis**

The lattice parameter analysis, based on lattice constant values of pristine BST and BST/WSe₂ heterostructures (Table S2), reveals varying strain degrees among the four samples. The values were averaged across multiple crystallographic orientations to ensure representative measurements. Samples S1 and S2 exhibit notable contraction along both the c- and a-axes, with average reductions of approximately 0.39% and 0.36% for S1, and 0.37% and 0.36% for S2, respectively. The uniform shrinkage along both lattice directions indicates compressive strain, likely from interfacial stress or defect incorporation during heterostructure formation. In contrast, S3 exhibits negligible contraction (0.01% along c, 0.02% along a), reflecting a nearly relaxed lattice with reduced distortion, possibly due to improved crystallinity or minimal interfacial mismatch. Sample S4 exhibits anisotropic strain behavior, with an average expansion along the c-axis (0.049%) and contraction along the a-axis (0.12%). This asymmetry implies local structural inhomogeneity, possibly caused by interfacial effects, isolated W atoms, or stacking faults, which perturb the lattice symmetry and lead to directionally dependent strain accommodation.

**Thermoelectric Transport Analysis**

Figures S5 to S10 collectively illustrate the influence of WSe₂ integration on the electronic transport and thermoelectric performance of BST-based heterostructures across samples S1 to S4. Figure S5 demonstrates the temperature dependence of mobility (log μ vs. log T), with slopes of –2.33 (S1), –2.9 (S2), –2.23 (S3), and –2.55 (S4). These negative slopes confirm the dominance of temperature-dependent scattering. Since the observed temperature dependence aligns more closely with acoustic phonon and defect scattering, and deviates from the behavior expected for ionized impurity or polar optical phonon scattering, these latter mechanisms can be ruled out, especially in S2 and S4.

Figure S6 presents the Seebeck effective mass $m_{s}^{*}$/mₑ, which varies across the samples: 0.31–0.59 (S1), 0.53–1.98 (S2), 0.53–0.7 (S3), and 0.52–1.31 (S4). The lower and more stable values in S1 and S3 suggest single-valley, nearly parabolic transport behavior. In contrast, the larger and more temperature-dependent $m_{s}^{*}$ observed in S2 and S4 reflect stronger band nonparabolicity and multivalley conduction, likely due to carrier excitation into higher energy bands. These features indicate more complex band structures and increased scattering in S2 and S4, consistent with their higher $m_{s}^{*}$ and steeper *μ* slopes.

Figure S8 presents the temperature-dependent electrical conductivity. In S1, pristine BST exhibits typical semiconducting behavior (104–222 S cm⁻¹), while doped BST shows a higher initial conductivity (~134 S cm⁻¹) due to interfacial carrier injection; however, it saturates early, with a crossover occurring near 330 K. This behavior reflects ambipolar conduction and increased interfacial scattering in the WSe₂ layer. In S2, BST shows weak metallic behavior followed by thermally activated transport (264 – 279 S cm⁻¹), whereas BST/WSe₂ displays a marked and steady increase (450 – 583 S cm⁻¹), indicating enhanced interfacial transport and possible band alignment effects. In S3 and S4, both BST and BST/WSe₂ exhibit decreasing conductivity with temperature, characteristic of degenerate semiconductors. However, BST/WSe₂ samples start with lower conductivity and follow similar or slightly divergent trends, likely due to defect-induced scattering (e.g., from isolated W atoms or stacking faults). The modest recovery in S4 suggests thermal activation of additional transport channels.

Figure S9 highlights Seebeck coefficient trends. S1 BST shows a monotonic decrease (~242 – 173 μV/K), typical of thermally activated carrier increase. In contrast, BST/WSe₂ displays a non-monotonic Seebeck trend (~168 – 220 – 187 – 192 μV K⁻¹), reflecting competing effects: energy filtering and favorable band alignment at low-to-mid temperatures, followed by bipolar diffusion and trap-state saturation^[61]^ at higher temperatures. In S2, BST gradually decreases (~340 – 274 μV/K) with a local maximum near 443 K, while BST/WSe₂ steadily increases (~151 – 323 μV/K), suggesting interfacial energy filtering and enhanced carrier transport. S3 shows similar trends in both variants, but the WSe₂-doped sample exhibits a sharper drop after the peak (~225 – 196 μV/K), likely due to increased scattering. In S4, BST/WSe₂ consistently outperforms BST, increasing linearly (~200 – 360 μV/K), indicating stable and efficient carrier transport across the heterointerface.

Figure S10 compares the power factor (PF) trends. In S1, BST maintains a nearly constant PF (~6.1–6.7 μW cm⁻¹ K⁻²), and BST/WSe₂ shows an initial increase (~3.8 – 7.2 μW cm⁻¹ K⁻²), a dip near 440 K, and recovery to ~7.3 μW cm⁻¹ K⁻², indicating thermally activated transport modulated by energy filtering. S2 exhibits the most significant improvement upon doping: BST shows a decline (~26 – 18 μW cm⁻¹ K⁻²), while BST/WSe₂ increases dramatically (~10 – 61 μW cm⁻¹ K⁻²), attributed to enhanced interfacial transport and ambipolar conduction. In S3, both systems exhibit similar PF trends, but BST/WSe₂ remains lower (~9–14 μW cm⁻¹ K⁻²), likely due to strong defect scattering. S4 reinforces the effectiveness of WSe₂ integration, with BST/WSe₂ exhibiting a two-stage increase (~10 – 35 μW cm⁻¹ K⁻²), in contrast to the modest rise and fall in pure BST.

In summary, WSe₂ incorporation into BST modulates transport properties via interfacial band alignment, ambipolar conduction, and defect scattering. This leads to improved thermoelectric performance in specific configurations, particularly S2 and S4, where favorable energy filtering and interfacial carrier dynamics significantly enhance the Seebeck coefficient and power factor.

**Table S1:** EDS data for samples deposited at different temperatures.

| Elements | Atomic% | | | | |  | | | |
| --- | --- | --- | --- | --- | --- | --- | --- | --- | --- |
|  | Bi M | Sb L | Te L | W M | Se L | Bi | Sb | Bi+Sb | Te |
| 573 K | 9.40 | 29.40 | 60.51 | 0.55 | 0.14 | 0.466039 | 1.45761 | 1.923649 | 3 |
| 623 K | 8.77 | 30.75 | 58.33 | 1.48 | 0.67 | 0.451054 | 1.581519 | 2.032573 | 3 |
| 673 K | 8.13 | 28.09 | 55.07 | 7.97 | 0.75 | 0.442891 | 1.530234 | 1.973125 | 3 |
| 723 K | 9.67 | 30.14 | 58.23 | 1.45 | 0.51 | 0.498197 | 1.552808 | 2.051005 | 3 |

**Table S2**: Crystallite size and dislocation densities of the four samples.

|  |  |  |  |  |  |  | Crystallite size | | Dislocation density | Average δ(nm^-2^) |
| --- | --- | --- | --- | --- | --- | --- | --- | --- | --- | --- |
| S1 | 2θ | 2θ/2=θ (deg) | θ (rad) | FWHM (deg) | FWHM, β (rad) | β cosθ | D = Kλ/β cosθ | Average D (nm) | δ=1/D^2^ (nm^-2^) |  |
|  | 17.53 | 8.77 | 0.15 | 0.20 | 0.003438124 | 0.0034 | 44.46778804 |  | 0.000505719 |  |
|  | 26.38 | 13.19 | 0.23 | 0.22 | 0.003752981 | 0.0037 | 41.35233485 |  | 0.00058479 |  |
|  | 28.12 | 14.06 | 0.25 | 0.21 | 0.003663795 | 0.0036 | 42.51515098 |  | 0.000553239 |  |
|  | 35.41 | 17.71 | 0.31 | 0.25 | 0.004341507 | 0.0041 | 36.53410043 |  | 0.000749209 |  |
|  | 38.20 | 19.10 | 0.33 | 0.25 | 0.004276057 | 0.0040 | 37.39500561 |  | 0.00071511 |  |
|  | 42.13 | 21.07 | 0.37 | 0.25 | 0.004288798 | 0.0040 | 37.75439762 |  | 0.00070156 |  |
|  | 44.68 | 22.34 | 0.39 | 0.29 | 0.005022883 | 0.0046 | 32.52344971 | 38.0641952 | 0.000945381 | 0.000718253 |
|  | 54.28 | 27.14 | 0.47 | 0.32 | 0.005559397 | 0.0049 | 30.54211614 |  | 0.001072017 |  |
|  | 58.16 | 29.08 | 0.51 | 0.28 | 0.004843987 | 0.0042 | 35.69273723 |  | 0.000784947 |  |
|  | 61.72 | 30.86 | 0.54 | 0.24 | 0.004204498 | 0.0036 | 41.86487144 |  | 0.000570559 |  |
|  |  |  |  |  |  |  |  |  |  |  |
| S2 | 17.5 | 8.75 | 0.15 | 0.19523 | 0.003407406 | 0.0034 | 44.86685599 |  | 0.000496762 |  |
|  | 26.38 | 13.19 | 0.23 | 0.20652 | 0.003604454 | 0.0035 | 43.05632656 |  | 0.000539419 |  |
|  | 28.12 | 14.06 | 0.25 | 0.22349 | 0.003900636 | 0.0038 | 39.93369052 |  | 0.000627077 |  |
|  | 35.41 | 17.705 | 0.31 | 0.23955 | 0.004180936 | 0.0040 | 37.9372051 |  | 0.000694815 |  |
|  | 38.23 | 19.115 | 0.33 | 0.24933 | 0.004351629 | 0.0041 | 36.74891644 |  | 0.000740476 |  |
|  | 42.13 | 21.065 | 0.37 | 0.21633 | 0.003775671 | 0.0035 | 42.88535168 | 36.34840742 | 0.000543728 | 0.000706208 |
|  | 44.65 | 22.325 | 0.39 | 0.27387 | 0.004779933 | 0.0044 | 34.17284489 |  | 0.000856323 |  |
|  | 54.25 | 27.125 | 0.47 | 0.30373 | 0.005301089 | 0.0048 | 32.02605994 |  | 0.000974974 |  |
|  | 58.12 | 29.06 | 0.51 | 0.29419 | 0.005134584 | 0.0045 | 33.66613482 |  | 0.000882294 |  |
|  |  |  |  |  |  |  |  |  |  |  |
| S3 | 17.5 | 8.75 | 0.15 | 0.20321 | 0.003546684 | 0.0035 | 43.10494708 |  | 0.000538203 |  |
|  | 26.35 | 13.175 | 0.23 | 0.22891 | 0.003995233 | 0.0039 | 38.84254651 |  | 0.000662803 |  |
|  | 28.12 | 14.06 | 0.25 | 0.2514 | 0.004387758 | 0.0043 | 35.50032018 |  | 0.000793479 |  |
|  | 35.43 | 17.715 | 0.31 | 0.24672 | 0.004306076 | 0.0041 | 36.83675421 |  | 0.000736949 |  |
|  | 38.44 | 19.22 | 0.34 | 0.29851 | 0.005209982 | 0.0049 | 30.71403266 | 34.63499644 | 0.00106005 |  |
|  | 42.04 | 21.02 | 0.37 | 0.23002 | 0.004014606 | 0.0037 | 40.32077795 |  | 0.000615095 | 0.000873345 |
|  | 44.65 | 22.325 | 0.39 | 0.28922 | 0.005047841 | 0.0047 | 32.35916268 |  | 0.000955005 |  |
|  | 51.4 | 25.7 | 0.45 | 0.29625 | 0.005170538 | 0.0047 | 32.43155279 |  | 0.000950746 |  |
|  | 54.25 | 27.125 | 0.47 | 0.31323 | 0.005466895 | 0.0049 | 31.05473673 |  | 0.001036918 |  |
|  | 58.09 | 29.045 | 0.51 | 0.31322 | 0.00546672 | 0.0048 | 31.61611621 |  | 0.001000421 |  |
|  | 61.81 | 30.905 | 0.54 | 0.35775 | 0.006243915 | 0.0054 | 28.20401381 |  | 0.001257124 |  |
|  |  |  |  |  |  |  |  |  |  |  |
| S4 | 17.5 | 8.75 | 0.152716 | 0.19039 | 0.003322932 | 0.0033 | 46.00743892 |  | 0.000472437 |  |
|  | 26.35 | 13.18 | 0.229947 | 0.19821 | 0.003459417 | 0.0034 | 44.85872217 |  | 0.000496943 |  |
|  | 28.09 | 14.05 | 0.245131 | 0.24638 | 0.004300142 | 0.0042 | 36.22126667 |  | 0.000762207 |  |
|  | 35.38 | 17.69 | 0.308749 | 0.23451 | 0.004092972 | 0.0039 | 38.74929994 |  | 0.000665997 |  |
|  | 38.23 | 19.12 | 0.33362 | 0.25727 | 0.004490209 | 0.0042 | 35.61475235 | 35.45566228 | 0.000788388 | 0.000747024 |
|  | 42.1 | 21.05 | 0.367392 | 0.22202 | 0.00387498 | 0.0036 | 41.78206037 |  | 0.000572823 |  |
|  | 44.62 | 22.31 | 0.389383 | 0.25515 | 0.004453208 | 0.0041 | 36.67611729 |  | 0.000743418 |  |
|  | 51.3 | 25.65 | 0.447677 | 0.27529 | 0.004804717 | 0.0043 | 34.88618443 |  | 0.000821662 |  |
|  | 54.19 | 27.10 | 0.472897 | 0.27747 | 0.004842765 | 0.0043 | 35.04763793 |  | 0.000814109 |  |
|  | 58.1 | 29.05 | 0.507018 | 0.30542 | 0.005330585 | 0.0047 | 32.42511915 |  | 0.000951123 |  |
|  | 61.87 | 30.94 | 0.539918 | 0.33901 | 0.005916841 | 0.0051 | 29.77242799 |  | 0.001128162 |  |

Note: K = 0.98 and λ = 0.154184 nm

**Table S3**: Lattice parameters of the four samples, BST/WSe₂ heterostructures and pristine BST, determined from different crystallographic orientations.

| c, a, b  (nm) | **S1**  c(00l) | a(0kl) | a(h0l) | a(hk0) | **S2**  c(00l) | a(0kl) | a(h0l) | a(hk0) | **S3**  c(00l) | a(0kl) | a(h0l) | a(hk0) | **S4**  c(00l) | a(0kl) | a(h0l) | a(hk0) |
| --- | --- | --- | --- | --- | --- | --- | --- | --- | --- | --- | --- | --- | --- | --- | --- | --- |
| (006) | 29.70 |  |  |  | 29.70 |  |  |  | 29.90 |  |  |  | 29.80 |  |  |  |
| (009) | 29.73 |  |  |  | 29.70 |  |  |  | 29.73 |  |  |  | 29.71 |  |  |  |
| (015) |  | 4.20 |  |  |  | 4.20 |  |  |  | 4.19 |  |  |  | 4.20 |  |  |
| (1010) |  |  | 4.21 |  |  |  | 4.19 |  |  |  | 4.191 |  |  |  | 4.19 |  |
| (110) |  |  |  | 4.19 |  |  |  | 4.19 |  |  |  | 4.19 |  |  |  | 4.19 |
| (0015) | 29.73 |  |  |  | 29.73 |  |  |  | 29.75 |  |  |  | 29.75 |  |  |  |
| (205) |  |  | 4.19 |  |  |  |  |  |  |  |  |  |  |  |  |  |
| (0018) | 29.74 |  |  |  | 29.73 |  |  |  | 29.74 |  |  |  | 29.74 |  |  |  |
| (0210) |  | 4.20 |  |  |  | 4.20 |  |  |  | 4.20 |  |  |  | 4.20 |  |  |
| (0021) | 29.73 |  |  |  | 29.73 |  |  |  | 29.58 |  |  |  | 29.69 |  |  |  |
| Pristine BST | | | | | | | | | | | | | | | | |
| (006) | 29.90 |  |  |  | 29.82 |  |  |  | 29.72 |  |  |  | 29.84 |  |  |  |
| (009) | 29.90 |  |  |  | 29.90 |  |  |  | 29.74 |  |  |  | 29.71 |  |  |  |
| (015) |  | 4.22 |  |  |  | 4.22 |  |  |  | 4.20 |  |  |  | 4.20 |  |  |
| (1010) |  |  | 4.23 |  |  |  | 4.24 |  |  |  | 4.20 |  |  |  | 4.21 |  |
| (110) |  |  |  | 4.20 |  |  |  | 4.20 |  |  |  | 4.19 |  |  |  | 4.19 |
| (0015) | 29.81 |  |  |  | 29.81 |  |  |  | 29.76 |  |  |  | 29.74 |  |  |  |
| (205) |  |  | 4.20 |  |  |  | 4.20 |  |  |  | 4.20 |  |  |  | 4.19 |  |
| (0018) | 29.80 |  |  |  | 29.80 |  |  |  | 29.75 |  |  |  | 29.72 |  |  |  |
| (0210) |  | 4.21 |  |  |  | 4.21 |  |  |  | 4.20 |  |  |  | 4.20 |  |  |

**Table S3:** Summary of Raman peaks in four specimens.

| Sample | Raman peaks (cm^-1^) | | | | | | |
| --- | --- | --- | --- | --- | --- | --- | --- |
|  | $A_{1g}^{1}$  Sb_2_Te_3_ | $A_{1g}^{2}$  SPM-Bi_2_​Te_3_ | $A_{1u}^{2}$  LSP- Bi_2_​Te_3_ | $A_{1g}^{2}$, $E_{g}$  Sb_2_Te_3_ | $E_{g}$, $B_{g}^{6}$  Sb_2_Te_3_ | $A_{1g}$  Sb_2_Te_3_ | $E_{2g}^{1}{,A}_{1g}$  WSe_2_ |
| S_1_ | 67.83 | 89.91 | 118.77 | 138.30 | 155.28 | - | 251.23 |
| S_2_ | 66.13 | 89.90 | 118.77 | 138.30 | 155.28 | 188.30 | 251.23 |
| S_3_ | 68.68 | 88.21 | 118.77 | 138.30 | 155.28 | 188.30 | 251.23 |
| S_4_ | 67.83 | 89.01 | 118.77 | 138.30 | 155.28 | 188.30 | 251.23 |


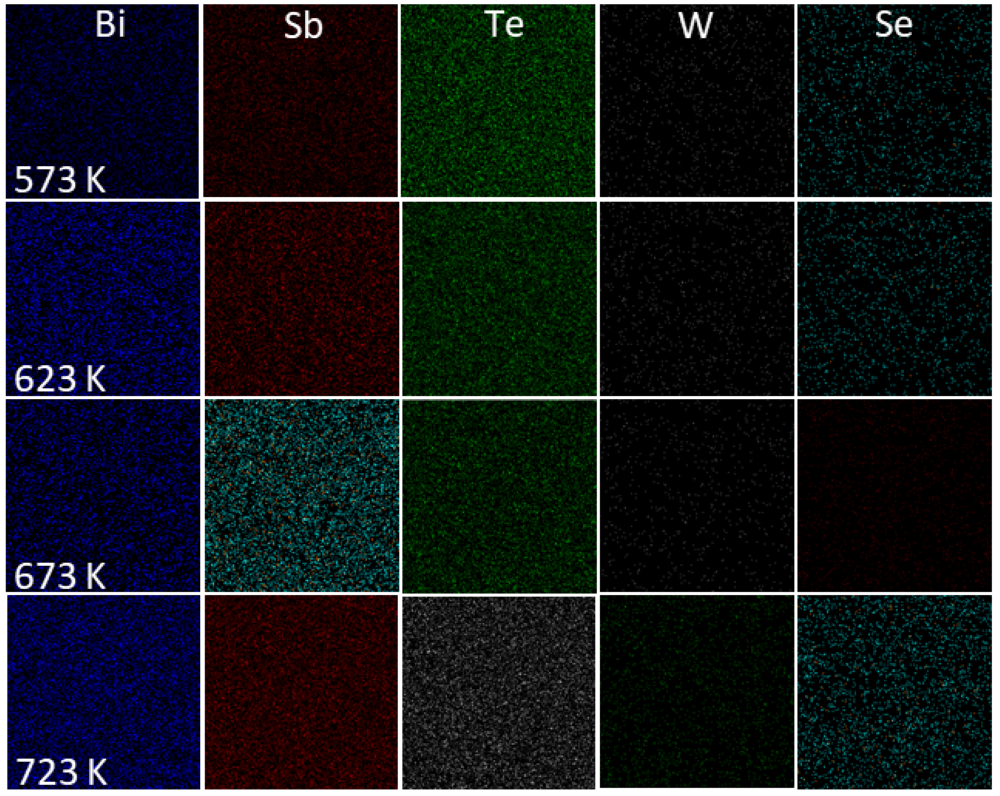


**Figure S1:** EDS mapping demonstrates the homogeneous distribution of constituent elements in the four samples.


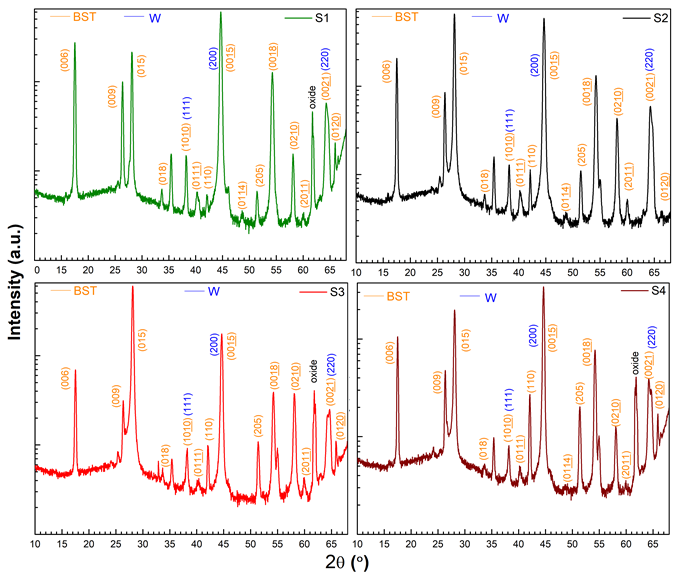
**Figure S2**: XRD patterns of the four BST/WSe₂ heterostructures.


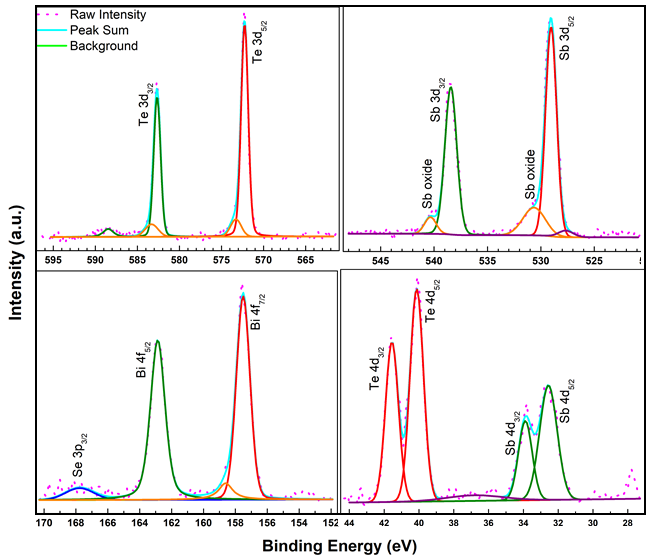


**Figure S3:** XPS spectra of BST/WSe₂ specimen constituents, including Te 3d, Sb 3d, Se 3p, Bi 4f, Te 4d, and Sb 4d, provide insights into the chemical states and bonding environment.


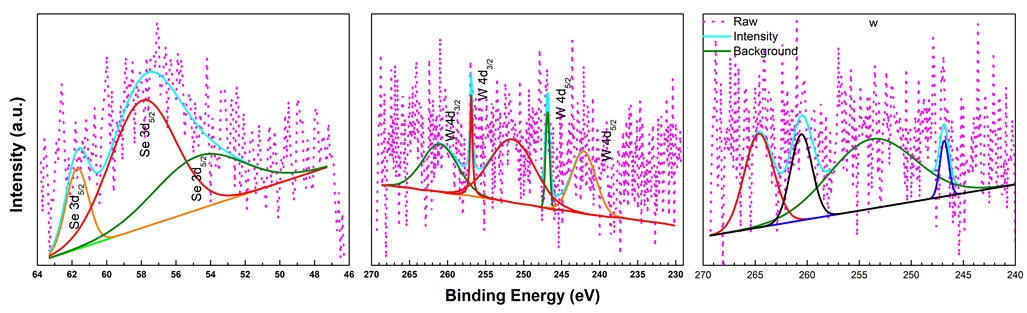
**Figure S4:** XPS spectra revealing characteristic peaks associated with Se and W in the BST/WSe₂ films.


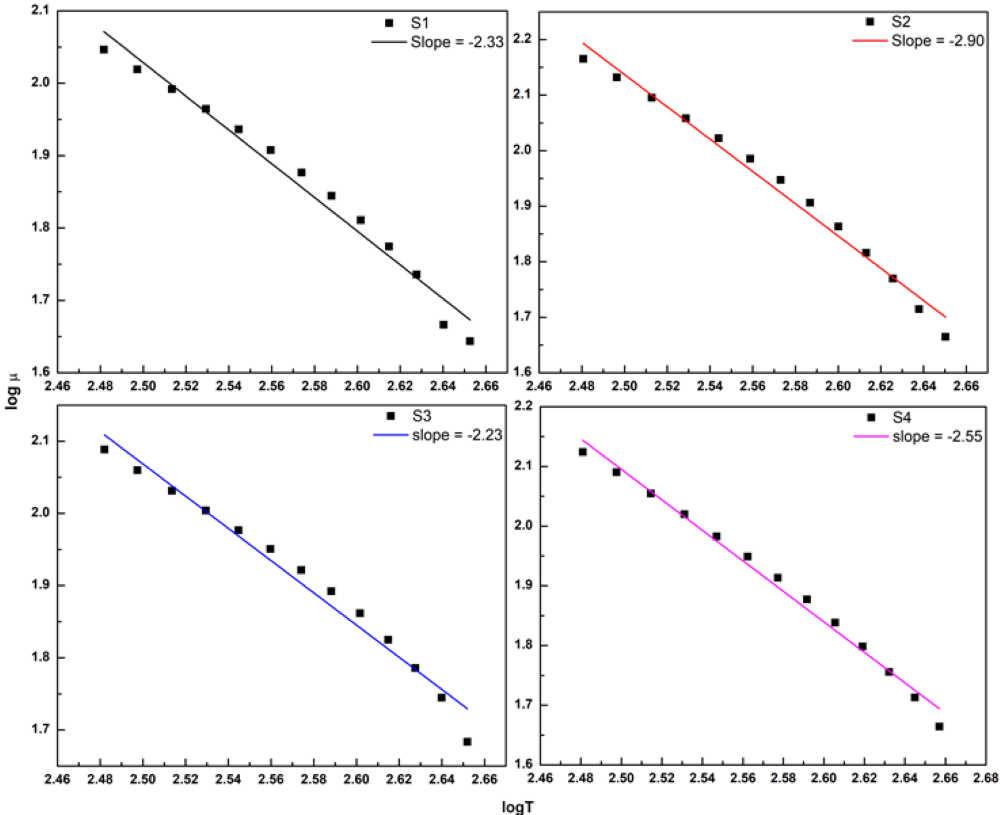
**Figure S5:** Logarithmic mobility plot versus temperature for slope extraction in four samples.


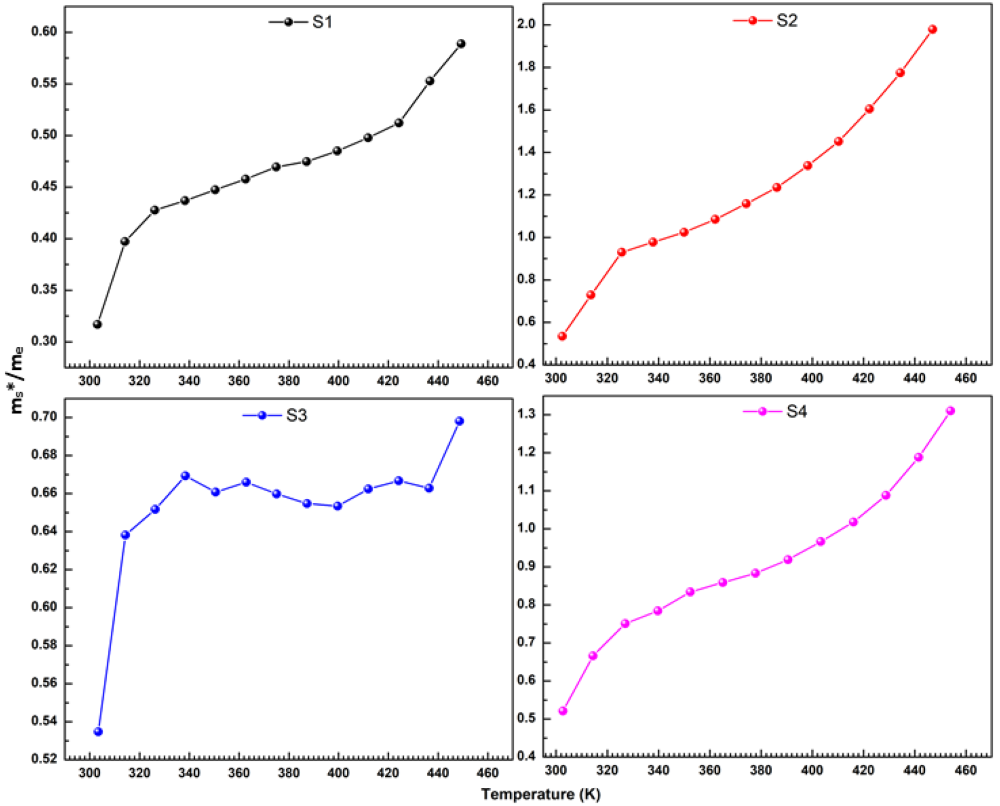
**Figure S6:** Seebeck effective mass as a function of temperature.


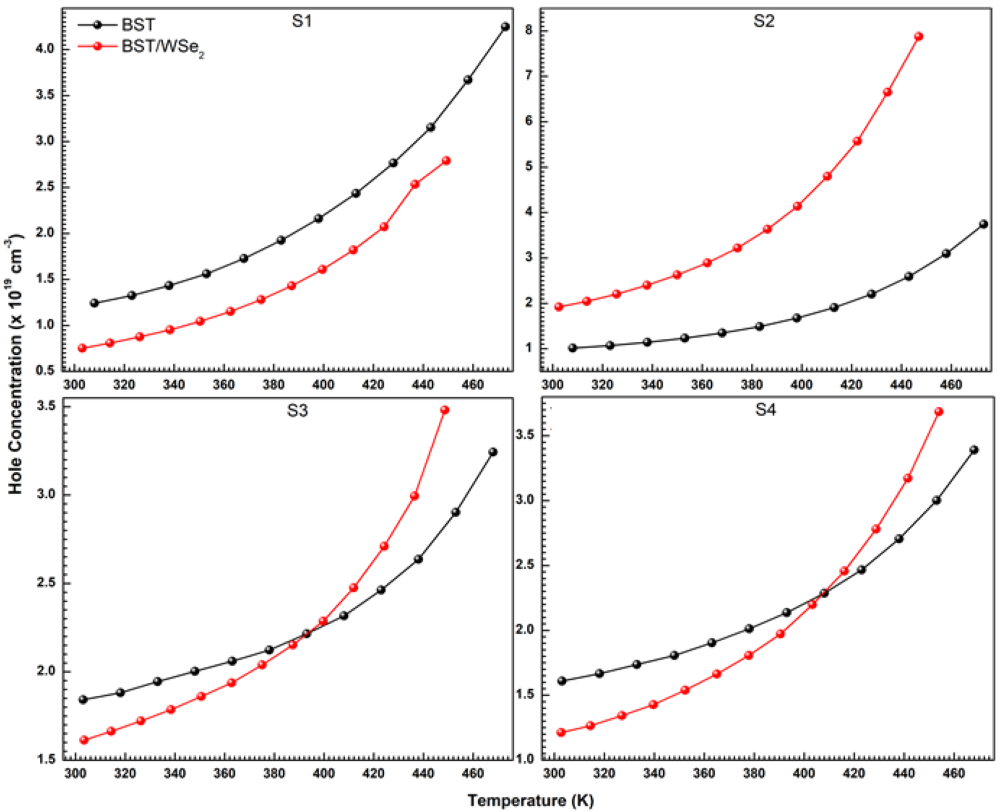


**Figure S7:** Comparison of the carrier concentration of the deposited samples with the corresponding pristine BST.


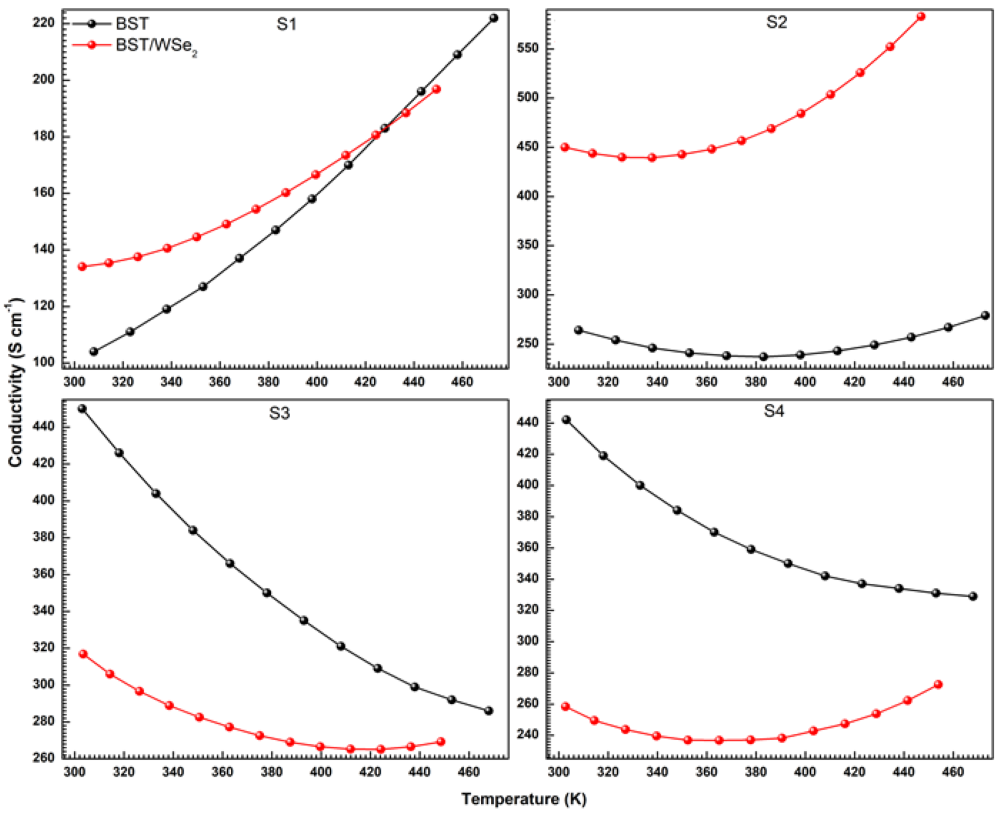


**Figure S8:** Comparison of the conductivity of the deposited samples with that of pristine BST.


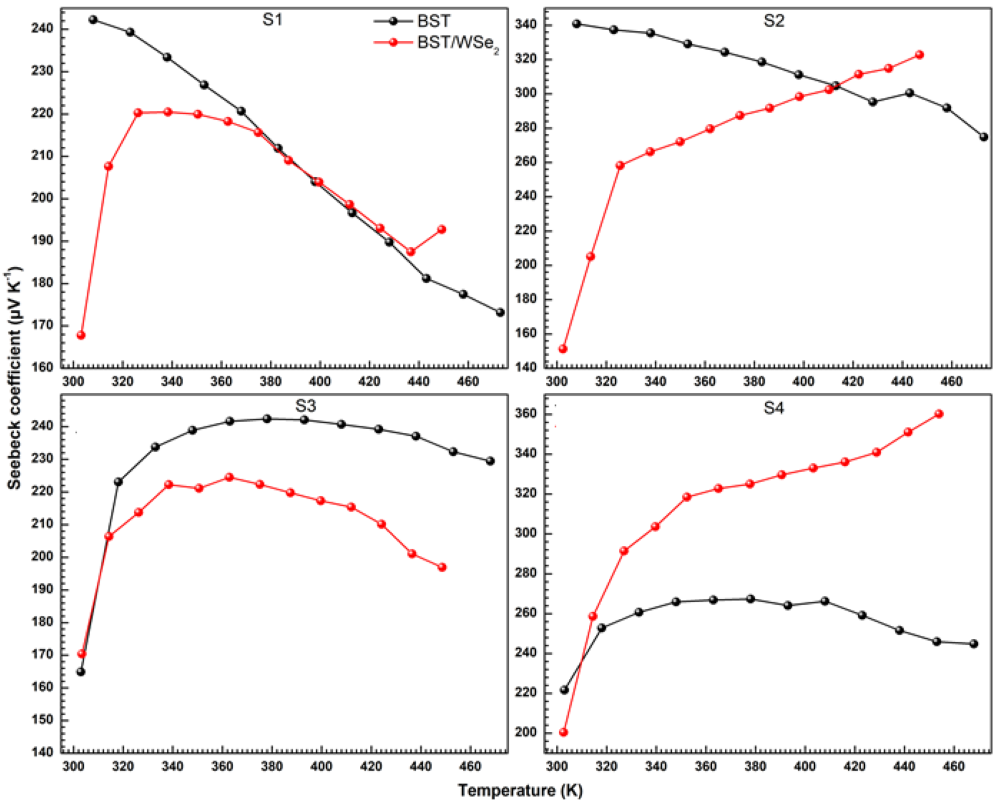


**Figure S9:** Comparison of the Seebeck coefficient of the heterostructures with pristine BST samples.


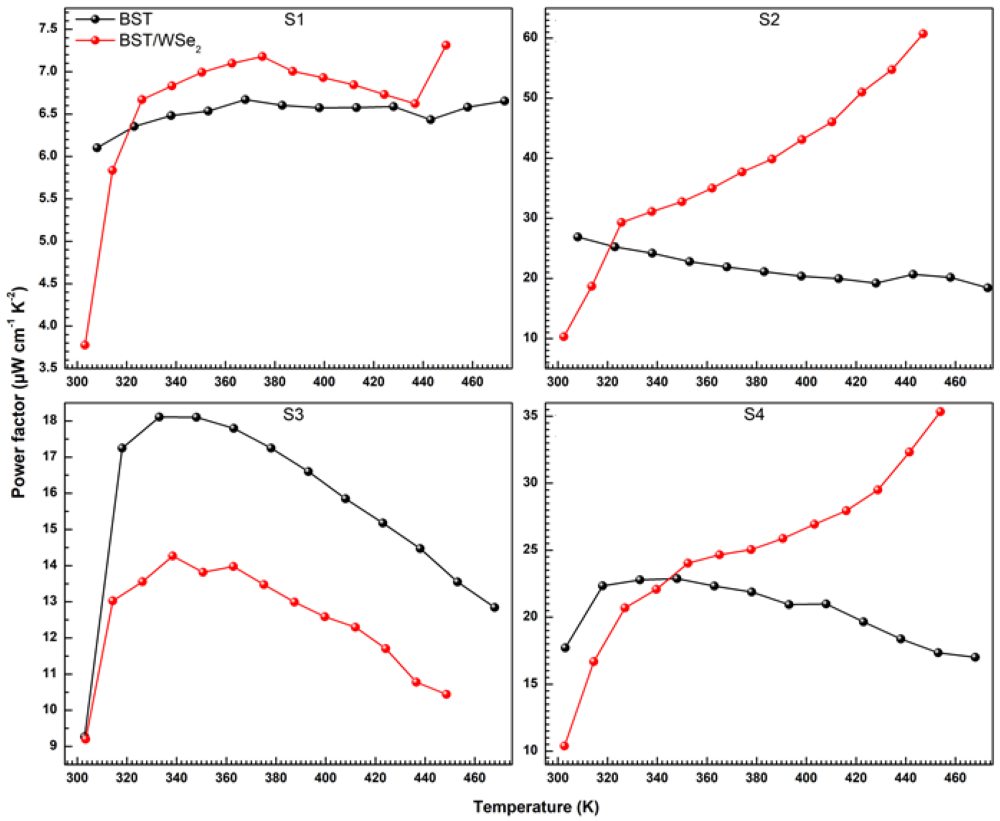


**Figure S10:** Comparison of the power factor of four samples with that of pristine BST samples.
